# Virological Outcomes of Second-line Protease Inhibitor–Based Treatment for Human Immunodeficiency Virus Type 1 in a High-Prevalence Rural South African Setting: A Competing-Risks Prospective Cohort Analysis

**DOI:** 10.1093/cid/cix015

**Published:** 2017-03-13

**Authors:** Dami Collier, Collins Iwuji, Anne Derache, Tulio de Oliveira, Nonhlanhla Okesola, Alexandra Calmy, Francois Dabis, Deenan Pillay, Ravindra K. Gupta, for the French National Agency for AIDS and Viral Hepatitis Research (ANRS) 12249 Treatment as Prevention (TasP) Study Group

**Affiliations:** 1 Department of Infection and Immunity, University CollegeLondon, United Kingdom; 2 Africa Health Research Institute, Durban, KwaZulu-Natal, South Africa; 3 Research Department of Infection and Population Health, University CollegeLondon, United Kingdom; 4 Sorbonne Universités, University Pierre and Marie Curie Université Paris 06, Inserm, Institut Pierre Louis d’épidémiologie et de Santé Publique (IPLESP UMRS 1136), Paris, France; 5 University of KwaZulu-Natal, Durban, South Africa; 6 Geneva University Hospital, HIV Unit, Department of Internal Medicine, Switzerland; 7 INSERM U1219—Centre Inserm Bordeaux Population Health, Université de Bordeaux, France; 8 Université de Bordeaux, ISPED, Centre INSERM U1219-Bordeaux Population Health, France

**Keywords:** HIV, antiretroviral therapy, virological failure, second line, protease inhibitor

## Abstract

**Background.:**

Second-line antiretroviral therapy (ART) based on ritonavir-boosted protease inhibitors (bPIs) represents the only available option after first-line failure for the majority of individuals living with human immunodeficiency virus (HIV) worldwide. Maximizing their effectiveness is imperative.

**Methods.:**

This cohort study was nested within the French National Agency for AIDS and Viral Hepatitis Research (ANRS) 12249 Treatment as Prevention (TasP) cluster-randomized trial in rural KwaZulu-Natal, South Africa. We prospectively investigated risk factors for virological failure (VF) of bPI-based ART in the combined study arms. VF was defined by a plasma viral load >1000 copies/mL ≥6 months after initiating bPI-based ART. Cumulative incidence of VF was estimated and competing risk regression was used to derive the subdistribution hazard ratio (SHR) of the associations between VF and patient clinical and demographic factors, taking into account death and loss to follow-up.

**Results.:**

One hundred one participants contributed 178.7 person-years of follow-up. Sixty-five percent were female; the median age was 37.4 years. Second-line ART regimens were based on ritonavir-boosted lopinavir, combined with zidovudine or tenofovir plus lamivudine or emtricitabine. The incidence of VF on second-line ART was 12.9 per 100 person-years (n = 23), and prevalence of VF at censoring was 17.8%. Thirteen of these 23 (56.5%) virologic failures resuppressed after a median of 8.0 months (interquartile range, 2.8–16.8 months) in this setting where viral load monitoring was available. Tuberculosis treatment was associated with VF (SHR, 11.50 [95% confidence interval, 3.92–33.74]; *P* < .001).

**Conclusions.:**

Second-line VF was frequent in this setting. Resuppression occurred in more than half of failures, highlighting the value of viral load monitoring of second-line ART. Tuberculosis was associated with VF; therefore, novel approaches to optimize the effectiveness of PI-based ART in high-tuberculosis-burden settings are needed.

**Clinical Trials Registration.:**

NCT01509508.

## Summary

A community based, second-line treatment cohort of 102 subjects was followed monthly in community clinics with a total of 178 patient-years of follow-up; second-line virological failure was frequent in this setting.

Despite clinical and public health gains following antiretroviral therapy (ART) rollout, treatment failure of first-line nonnucleoside reverse transcriptase inhibitor (NNRTI)–based ART is common [1–4], with up to 3 million human immunodeficiency virus (HIV)–infected patients estimated to receive second-line, boosted protease inhibitor (bPI)–based ART by 2020 [5]. Treatment failure on second-line ART is a major concern given poor, if any, access to further regimens in high-burden settings.

Data from observational studies of second-line bPI-based ART treatment outcomes in sub-Saharan Africa suggest a 14%–32% prevalence of virological failure (VF) [6–12], with randomized trials from comparable settings reporting lower prevalence of VF at 17%–19% at 48 weeks and longer durations of follow-up [13–16]. These studies were largely conducted in urban or periurban areas. Although associations have been reported between second-line VF and poor adherence [6, 8, 10, 17, 18] and delayed switch [7, 9], socioeconomic and demographic factors such as education level, employment, and data on household members have not been studied together. Furthermore, the potentially critical association between concomitant tuberculosis (TB) [17] and second-line bPI failure is inconclusive.

Previously published cohort studies have used a Kaplan-Meier survival analysis and standard Cox regression models that can result in inflated estimates due to competing events. Competing risk regression analysis overcomes this limitation by accounting for events, such as death, that preclude the subject from experiencing the study outcome. This is particularly important in sub-Saharan Africa where mortality remains significant following ART initiation due to advanced disease stage as well as loss to follow-up [19]. Here we have applied competing risk methods to comprehensively and prospectively investigate risk factors for VF of bPI-based second-line ART in a rural population of South Africa. In this setting ART was readily available and follow-up optimum according to local standards, including real-time viral load monitoring.

## MATERIALS AND METHODS

### Study Setting and Study Design

This cohort study was nested within the French National Agency for AIDS and Viral Hepatitis Research (ANRS) 12249 Treatment as Prevention (TasP) trial (ClinicalTrials.gov identifier: NCT01509508), an ongoing cluster-randomized trial evaluating the impact of immediate vs deferred ART initiation (according to South African guidelines) on HIV incidence [20] (see Supplementary Materials). The trial started recruiting in March 2012 in Hlabisa subdistrict, Umkhanyakude district, Northern KwaZulu-Natal, where the antenatal prevalence of HIV is 44%, one of the highest in South Africa [21, 22]. This rural setting is also one of the poorest districts in South Africa with very high unemployment rates.

This analysis is based on the treatment outcomes documented prospectively in the subgroup of patients on second-line bPI-based treatment within the TasP trial. Participants from all clusters irrespective of trial arm were included in this cohort. Participants were aged >15 years, resident in Hlabisa subdistrict, and included from the date of initiation of second-line treatment till the earliest of the date last seen in clinic, death, or loss to follow-up. No participants initiating bPI ART after May 2015 were included to allow at least 6 months’ follow-up for the primary outcome to occur. The participants’ outcomes were recorded until November 2015.

### Outcome and Prespecified Explanatory Variables

The primary outcome was VF defined as a VL >1000 copies/mL on at least 1 occasion ≥6 months after commencing second-line treatment. Any death occurring during the trial period was recorded and follow-up time was censored at the date of death. Loss to follow-up was defined as ≥3 consecutive missed clinic appointments. Follow-up time was censored at the last clinic visit. For all the other participants who remained in the trial with virological suppression (VL < 1000 copies/mL), follow-up was censored as the latest of either the date of the last laboratory test or last clinic visit.

### Clinic Procedures and Laboratory Methods

At presentation to the trial clinics, all consenting HIV-infected participants were asked to complete the study questionnaires and underwent clinical examination by trial nurses. CD4 count was measured in TasP clinics using a commercial point-of-care CD4 test (Alere PIMA device tool, Alere Inc, Waltham, Massachusetts). VL was measured using The Abbott m2000 RealTime System with HIV type 1 (HIV-1) VL determination from human plasma of HIV-1–infected individuals in the range of 40–10000000 copies/mL (Abbott Molecular Inc, Des Plaines, Illinois). The viral load assay was CE (Conformité Européene) marked and performed at the Africa Centre laboratory; the laboratory participated in Quality Control for Molecular Diagnostics (QCMD) for VL quality assurance testing. Both CD4 and viral load were measured at baseline, and at months 3 and 6 after ART initiation and every 6 months thereafter. Full-genome deep sequencing was performed after 2 consecutive VL measurements >1000 copies/mL at least 6 months apart following second-line VF, as per adapted protocol from Gall et al [23]. In brief, 4 overlapping fragments, spanning approximately 9 kb of the HIV genome, were amplified and purified as per adapted protocol from Gall et al [23]. The library preparation was done on equimolar pooled amplicons, using the Nextera XT Library preparation kit, followed by sequencing on the Illumina MiSeq platform. Next-generation sequencing (NGS) data were analyzed on Geneious software and a threshold of 2% was used for minority variants detection, with a minimum coverage of 1000 reads. In the case of a single VL >1000 copies/mL, Sanger sequencing was done following Manasa et al’s protocol [24]. The external quality assurance for Sanger sequencing was with QCMD. Drug resistance mutations were identified according to the Stanford HIV Drug Resistance Database (http://hivdb.stanford.edu/) [25]. We reported mutations where they were detected above 2% frequency. Safety monitoring blood samples were also taken as per protocol [20].

### Statistical Analysis

Participant characteristics were reported using frequency and percentage for categorical variables and median and interquartile range (IQR) for continuous variables. The incidence of second-line failure per 100 person-years was estimated with its 95% confidence interval (CI).

We estimated the cumulative incidence function of VF on second-line treatment, taking into account death and loss to follow-up as competing risks. Competing risk regression was used to estimate the subdistribution hazard ratio (SHR) of the associations between VF and participant clinical and demographic factors, accounting for the competing risks of death and loss to follow-up, according to the model of Fine and Gray [26]. In the final multivariable model, mutually adjusted estimates of the SHRs were determined by including those factors with evidence of an association in the univariable analysis and a *P* value of <.1. Although age and sex were not significantly associated with VF in the univariable analysis, they were kept in the final model as they were a priori specified confounders. Analysis was done using Stata software version 13.

### Ethical Committee Approval

Ethics approval was granted by the Biomedical Research Ethics Committee of the University of KwaZulu-Natal (BFC 104/11) and the Medicines Control Council of South Africa. The study was also authorized by the KwaZulu-Natal Department of Health in South Africa. Written informed consent was obtained from all participants.

## RESULTS

One hundred one participants were included in this analysis. Sixteen (15.8%) individuals were already on second-line treatment at enrollment into the trial for a median of 2.7 years (IQR, 1.1–3.9 years). Three of these participants had VF at the baseline clinic visit. Seven (6.9%) participants had not initiated ART at enrollment into the trial. The remaining 78 (77.2%) were on first-line NNRTI-based ART for a median duration of 4.9 years (IQR, 3.2–6.7 years) at the time of enrollment; 41 (52.6%) of these had VF at the baseline clinic visit in the TasP trial, necessitating a switch to bPI along with 2 nucleoside reverse transcriptase inhibitors (NRTIs). Median duration on bPI for the 85 patients was 0.6 years (IQR, 0.3–0.9 years).

The majority of participants were female (65.4%) and the median age at initiation of second-line treatment was 37.4 years (IQR, 31.6–45.3 years). There was a high level of unemployment (91.9%) in this cohort of participants residing in a rural setting (Table 1). Thirteen individuals (12.9%) were diagnosed with TB during the study period.

**Table 1.** Demographic and Clinical Characteristics of Study Participants

Characteristic	No. (%)
Sex (n = 101)	
Female	66 (65.4)
Age at initiating bPI-based ART, y, median (IQR) (n = 101)	37.4 (31.6–45.3)
Relationship status (n = 100)	
Single	76 (76.0)
Married	17 (17.0)
Widowed	7 (7.0)
Employed (n = 87)	
Yes	7 (8.1)
Education level (n = 101)	
Primary or less	46 (45.5)
Some secondary	30 (29.7)
Completed secondary	25 (24.8)
Household asset ownership index score (n = 100)	
Low	37 (37.0)
Medium	52 (52.0)
High	11 (11.0)
Other HIV-positive household member (n = 101)	
Yes	68 (67.3)
Distance to national highway, km, median (IQR) (n = 101)	2.5 (1.4–5.6)
Distance to clinic, km, median (IQR) (n = 101)	1.2 (0.8–1.9)
Clinical characteristics	
Duration of HIV diagnosis, y, median (IQR) (n = 95)	5.1 (2.7–7.6)
Duration on NNRTI-based first-line ART, y, median (IQR) (n = 101)	4.6 (2.2–6.4)
Duration on bPI-based second-line ART, y, median (IQR) (n = 101)	2.0 (1.4–2.5)
On bPI before recruitment to TasP (n = 101)	
Yes	16 (15.8)
CD4 within 6 mo prior to switch to bPI, cells/mm^3^, median (IQR) (n = 31)	180 (107–343)
Viral load within 6 mo of switch to bPI, copies/mL (n = 61)	
<1000	18 (29.5)
>1000	43 (70.5)
Nadir CD4 count prior to first-line ART, cells/mm^3^, median (IQR) (n = 94)	95.5 (17.0–191.0)
Tuberculosis treatment within 6 mo of PI failure (n = 94)	5 (5.3)
No. of clinic visits/y, median (IQR) (n = 101)	12.7 (10.4–14.0)
WHO stage (n = 90) at cohort baseline	
1	47 (53.2)
2	18 (20.0)
3	23 (25.6)
4	2 (2.2)
Median pill count, % (n = 92)	
0–96	25 (27.2)
≥97	67 (72.8)
First-line regimen	
ZDV/d4T + 3TC/FTC + NVP/EFV	54 (53.5)
TDF + 3TC/FTC + NVP/EFV	47 (46.5)
Second-line regimen	
ZDV/TDF + 3TC/FTC + LPV/r	101 (100.0)

Data are presented as No. (%) unless otherwise indicated.

Abbreviations: 3TC, lamivudine; ART, antiretroviral therapy; bPI, ritonavir-boosted protease inhibitor; d4T, stavudine; EFV, efavirenz; FTC, emtricitabine; HIV, human immunodeficiency virus; IQR, interquartile range; LPV/r, lopinavir/ritonavir; NNRTI, nonnucleoside reverse transcriptase inhibitor; NVP, nevirapine; TasP, treatment as prevention; TDF, tenofovir disoproxil fumarate; WHO, World Health Organization; ZDV, zidovudine.

### Virological Failure and Associated Risk Factors

The 101 participants contributed 178.7 person-years of follow-up to the analysis. The overall incidence of VF on second-line ART was 12.9 per 100 person-years. At administrative censoring, 76 participants were alive and in care, 1 had died, and 1 was lost to follow-up before any VF was documented, and 23 participants had VF at least 6 months after initiating bPI second-line ART (Supplementary Figure 1). Following intensification of adherence counseling, 13 of the 23 participants (56.5%), including the 3 patients with bPI VF at the baseline clinic visit, resuppressed with a VL <1000 copies/mL after a median of 8.0 months (IQR, 2.8–16.8 months) (Supplementary Figure 2). Eight of these 13 participants subsequently rebounded with a VL >1000 copies/mL. The prevalence of VF in patients alive and on bPI ART at the time of administrative censoring was 17.8% (18/101).

In the univariable analysis, second-line VF was associated with concomitant TB treatment within 6 months of failure (SHR, 15.9 [95% CI, 6.2–40.6]; *P* < .0001) and a lower level of adherence (median pill count <97%) (SHR, 2.4 [95% CI, 1.0–5.7]; *P* = .04). In the multivariable analysis, the association of TB treatment with VF on bPI second-line treatment remained (SHR, 11.5 [95% CI, 3.9–33.7]; *P* < .001), whereas the association with median pill count was no longer present (Table 2).

**Table 2.** Subdistribution Hazard Ratios (SHRs) of Clinical and Demographic Characteristics and Association With Virological Failure on Second-line Ritonavir-Boosted Protease Inhibitor–Based Treatment: Univariable Analysis Followed by Multivariable Model of Mutually Adjusted SHRs

Characteristic	Univariable Model				Multivariable Model	
	Events/Follow-up Time^a^	Rate (95% CI)^b^	SHR (95% CI)	*P* Value	SHR (95% CI)	*P* Value
Sex						
Female	15/1.29	11.59 (6.99–19.22)	1	.90	1	.79
Male	8/0.51	15.72 (7.87-31.45)	1.06 (.45–2.49)		0.85 (.18–1.64)	
Age at initiating bPI-based ART, y						
16–35	11/0.78	14.18 (7.85–25.61)	1	.61	1	.98
≥35	12/1.03	11.68 (6.63–20.56)	0.81 (.36–1.82)		1.02 (.33–3.04)	
Relationship status						
Single	18/1.34	13.48 (8.49–21.39)	1	.55		
Married	3/0.34	8.68 (2.80–26.90)	0.72 (.19–2.67)			
Widowed	2/0.10	20.20 (5.05–80.75)	1.72 (.53–5.59)			
Education level						
Primary or less	11/0.84	13.13 (7.27–23.71)	1	.93		
Some secondary	6/0.43	13.85 (6.22–30.83)	1.16 (.47–2.84)			
Completed secondary	6/0.53	11.28 (5.07–25.10)	1.18 (.41–3.38)			
Employed						
No	16/1.29	12.41 (7.60–20.26)	1	.87		
Yes	1/0.09	10.65 (1.50–75.62)	0.86 (.16–4.79)			
Household asset ownership index score						
Low	7/0.69	10.20 (4.86–21.39)	1	.87		
Medium	14/0.92	15.18 (8.99–25.63)	1.14 (.44–2.93)			
High	2/0.17	11.55 (2.89–46.20)	1.42 (.37–5.47)			
Other HIV-positive household member						
No	8/0.56	14.29 (7.14–28.57)	1	.70		
Yes	15/1.24	12.07 (7.27–20.12)	1.17 (.52–2.61)			
Distance to national highway, km						
<2	14/0.69	20.35 (12.05–34.36)	1	.16		
2–16	9/1.12	8.07 (4.20–15.51)	0.54 (.23–1.27)			
Distance to clinic, km						
<1	10/0.76	13.23 (7.12–24.58)	1			
1–2	8/0.61	13.11 (6.55–26.20)	1.78 (.70–4.52)			
2–4	5/0.44	11.45 (4.77–27.52)	0.81 (.27–2.41)	.34		
Clinical characteristics						
Nadir CD4 prior to first-line ART, cells/mm^3^						
<100	8/0.48	16.78 (8.39–33.56)	1	.37		
≥100	10/0.93	10.72 (5.77–19.92)	0.66 (.26–1.66)			
Tuberculosis treatment within 6 mo of PI failure						
No	17/1.62	10.47 (6.51–16.84)	1	<.0001^c^	1	<.001^c^
Yes	4/0.03	116.23 (43.62–309.68)	15.86 (6.21–40.56)		11.50 (3.92–33.74)	
No. of visits per year						
0–11	17/0.91	18.76 (11.67–30.18)	1	.75		
12–22	6/0.90	6.69 (3.01–14.89)	0.85 (.30–2.34)			
WHO stage						
1	7/0.80	8.79 (4.19–18.44)	1	.26		
2	7/0.26	27.12 (12.93–56.89)	2.03 (.86–4.81)			
3/4	7/0.50	13.94 (6.64–29.24)	1.37 (.42–4.51)			
Median pill count, %						
≥97	12/1.30	27.14 (14.12–52.16)	1	.04^c^	1	.28
0–96	9/0.33	9.20 (5.23–16.20)	2.41 (1.02–5.65)		1.83 (0.61–5.50)	
Duration between HIV diagnosis and baseline. y						
0–3	8/0.66	12.21 (6.11–24.42)	1	.87		
4–7	8/0.64	12.41 (6.21–24.82)	0.80 (.31–2.05)			
8–20	5/0.36	13.75 (5.73–33.05)	1.01 (.32–3.17)			
On PI before recruitment to TasP						
No	16/1.31	12.22 (7.49–19.95)	1	.13		
Yes	7/0.49	14.17 (6.76–29.73)	1.97 (.81–4.75)			
Duration on first-line regimen, y						
<3	7/0.61	11.50 (5.48–24.12)	1	.35		
3–5	7/0.73	9.58 (4.67–20.09)	0.69 (.24–1.94)			
6–12	9/0.46	19.42 (10.11–37.33)	1.38 (.50–3.84)			
Duration on second-line regimen, y						
<2	8/0.56	14.18 (7.09–28.36)	1	.72		
2–3	9/0.70	12.87 (6.70–24.73)	0.99 (.38–2.56)			
3–10	6/0.54	11.12 (5.00–24.75)	1.47 (.49–4.40)			

Data are presented as No. (%) unless otherwise indicated.

Abbreviations: ART, antiretroviral therapy; bPI, ritonavir-boosted protease inhibitor; CI, confidence interval; HIV, human immunodeficiency virus; PI, protease inhibitor; SHR, subdistribution hazard ratio; TasP, treatment as prevention; WHO, World Health Organization.

a Follow-up time in 100 person-years.

b Rate per 100 person-years.

c Associations with some evidence against the null.

Thirteen participants were diagnosed with TB and received antituberculosis treatment during the study observation period; 4 of them were treated within 6 months of VF; 1 participant, within 6 months of censoring, did not experience VF; the remaining 8 participants were diagnosed and treated for TB at time points distant from their study exit and did not exhibit VF after initiation of antituberculosis treatment.

### Drug Resistance

Genotypes were available at first-line NNRTI ART failure in 9 participants and at bPI ART failure in 6 participants of the 23 with VF failure of second-line ART (Table 3). The reasons for missing genotypes were as follows: 4 patients receiving care in Department of Health clinics and therefore no sample was available; resuppression in 10 patients and therefore no viremic confirmatory sample available for genotyping; and no confirmatory sample in 2. Three of 5 patients exposed to tenofovir in their first-line regimen developed high-level tenofovir resistance with the K65R mutation, and 2 had accessory tenofovir mutations A62V, V75I, or F77L in the reverse transcriptase gene. In one of these individuals, K65R was detected only by NGS (at 12% frequency; Table 3). All first-line failures had major NNRTI resistance and 5 of 9 had high-level lamivudine/emtricitabine resistance (M184V/I). An additional drug resistance–associated variant (at <20% frequency) conferring NNRTI resistance was detected in patient 6 (K103N at 12%). NGS did not detect minority variants in the protease gene in any of the 9 patients with second-line failure.

**Table 3.** Resistance Mutations Identified by Next-Generation Sequencing

Participant ID	First-line Regimen	Second-line Regimen	Duration on bPI, y	Time-point	PI Mutations	NRTI Mutations	NNRTI Mutations
1	d4T, 3TC, EFV	TDF, 3TC, LPV/r	2.0	First-line failure	—	M184V	K103N, P225H, K238T
				Second-line failure	—	—	*P225H*_10%_, *K238T*_8%_
2	TDF, FTC, EFV	ZDV, 3TC, LPV/r	2.3	First-line failure	—	—	—
				Second-line failure	—	—	—
3	TDF, 3TC, EFV	TDF, 3TC, LPV/r	1.1	First-line failure	—	A62V, K65R, V75I, Y115F	E138Q, G190E
				Second-line failure*	—	—	—
4	d4T, 3TC, NVP d4T, 3TC, EFV	TDF, 3TC, LPV/r	1.7	First-line failure	—	M184V	K103N, P255H
				Second-line failure	—	—	—
5	d4T, 3TC, EFV	TDF, 3TC, LPV/r	1.9	First-line failure	—	T69N, K70N	V106M, *E138G*_6%_, G190A, F227L
				Second-line failure	—	—	
6	TDF, 3TC, EFV	ZDV, 3TC, LPV/r	2.1	First-line failure	—	—	*K103N*_12%_, V106M, G190A
				Second-line failure	—		
7	d4T, 3TC, EFV ZDV, 3TC, EFV	TDF, 3TC, LPV/r	1.6	First-line failure	—	M41L, L74I, V75L, M184V, T215Y	V106M, V179D
				Second-line failure	—		—
8	d4T, 3TC, EFV TDF, 3TC, EFV	ZDV, 3TC, LPV/r	2.0	First-line failure	—	M41L, A62V, K65R, *K70T*_2%_, V75I, M184V	K103N, V106M, E138G, *F227L*_6%_
				Second-line failure	**M46I, I54V, L76V, V82A**	**T215Y**	*E138G*_5%_
9	d4T, 3TC, NVP TDF, FTC, EFV	TDF, 3TC, LPV/r	1.5	First-line failure	—	*K65R*_12%_, M184V	K103N, Y188L/*F*_15%_, K238T
				Second-line failure	—		

First-line failure time-point indicates mutations present at first-line NNRTI virological failure. Second-line failure time-point indicates mutations acquired or lost where there are second-line bPI failure sequencing data available. These are presented in boldface type where they are newly acquired and where a mutation is lost. Minority variants detected between 2% and 20% are reported in italic type with their respective frequencies in subscript. Sequences with an asterisk (*) indicate population sequencing data derived by Sanger methodology. Dash (—) indicates no mutations.

Abbreviations: 3TC, lamivudine; bPI, ritonavir-boosted protease inhibitor; d4T, stavudine; EFV, efavirenz; FTC, emtricitabine; ID, Identity Document; LPV/r, lopinavir/ritonavir; NNRTI, nonnucleoside reverse transcriptase inhibitor; NRTI, nucleoside reverse transcriptase inhibitor; NVP, nevirapine; PI, protease inhibitor; TDF, tenofovir disoproxil fumarate; ZDV, zidovudine.

At second-line bPI ART failure, only 1 of 8 (12.5%) participants had acquired major PI mutations: M46I, I54V, L76V, and V82A. This individual received rifampicin containing TB treatment started at the same time as double-dose bPI.

## DISCUSSION

We determined the incidence rate for VF on second-line ART and associated risk factors in rural KwaZulu-Natal within the TasP trial. The incidence rate of VF was 12.9 per 100 person-years (95% CI, 8.6–19.4), and prevalence was 17.8% at the end of the observation period. A meta-analysis of studies conducted in resource-limited settings reported a pooled prevalence of VF of 23.1% (range, 11.4%–39.9%) after 12 months of treatment with bPI ART [6], although our prevalence estimate was based on a follow-up period of <1 year in those initiated on second-line ART within TasP. More recent randomized trials reported a lower prevalence of VF, between 14% and 19% at 48 and 96 weeks of treatment [13–15, 27]. However, the lower prevalence within trials may not be generalizable to the “real world” because trial participants are closely monitored.

There are considerable differences between studies in the definition of VF (from VL >50 to >1000 copies/mL), and guidelines are not clear on a definition of second-line failure that should trigger a switch of treatment regimen. We therefore chose a pragmatic definition of a single VL >1000 copies/mL, particularly as the follow-up time was relatively short for patients in TasP who switched to second-line ART within the trial. We found that many patients with VF on bPI ART resuppressed to VL <1000 copies/mL following a period of intense adherence counseling. This phenomenon has been reported in patients treated with first-line ART in both South Africa [28] and other parts of sub-Saharan Africa [29], and demonstrates that the efficacy of second-line treatment could be optimized if regular VL monitoring is widely available, adding to the impetus for development of point-of-care VL testing in tandem with effective adherence counseling.

The association between TB treatment and VF of NNRTI-based first-line ART has previously been reported [17]. Here we found evidence of an association between TB treatment and VF of second-line treatment. Our study design did not allow us to infer causality between TB treatment and VF, as some patients were never virologically suppressed even before the rifampicin treatment. TB disease itself could be a marker of virological and clinical failure; indeed, the 2010 World Health Organization (WHO) clinical criteria for treatment failure include a new diagnosis of TB [30]. In patients failing during the rifampicin treatment, the mechanism leading to VF may be due to drug interactions between PIs and rifampicin. Rifampicin, a potent cytochrome P450 3A4 inducer, significantly reduces the serum levels of PIs, and concurrent use can lead to VF [31, 32]. To compensate for this phenomenon, double-dosed bPI has been proposed—from twice-daily 400 mg/100 mg to 800 mg/200 mg of lopinavir/ritonavir, as is in this clinical setting [33]. However, the success of such a strategy may be limited by intolerance to the higher doses of PIs [34–36]. Nonadherence to treatment may also contribute to failure as the inherent polypharmacy required to treat both conditions may be a challenge for patients.

Poor adherence has been shown to be associated with VF in other studies [6, 8, 10, 17, 18]; however, we found no association between adherence measured by pill count and VF. This might in part be due to the fact that adherence was high in this trial context, although announced pill count is susceptible to pill dumping by participants and therefore not be a particularly good marker for adherence: indeed, 88% of participants on first-line NNRTI ART had an overall adherence of ≥95% at 6 months [37].

We found multiple major NRTI and NNRTI mutations at first-line failure in the participants who had genotype testing (9 participants), consistent with data from resource-limited settings [2, 38]. Though numbers were small, NGS increased detection of significant tenofovir resistance (K65R mutation) by around 50%, consistent with previous NGS studies in this setting [39]. We found acquired major protease mutations in only 1 (11%) failing bPI ART, consistent with other data from South Africa [8, 18, 40–42]. NGS did not increase the detection rate of mutations in protease. The high genetic barrier of bPI to resistance development could be a reason for this. It is worth noting that standard genotype tests based on *pol* sequencing ignore the influence of mutations in other genes such as *gag* [43–47] and *env* [48] on PI resistance. Notably, the individual with major protease resistance had received double-boosted PI treatment, and coupled with previous reports of multiple major protease resistance mutations in children treated with double-dose PI [49, 50], further work regarding this approach is warranted.

The main methodological strength of this study is the application of regression methods, which account for the presence of competing risks to estimate the rate of VF on second-line treatment and the association between covariates of interest and VF. Other cohort studies reporting outcomes on second-line treatment and factors associated with VF have used standard Kaplan-Meier survival analysis and Cox regression models, which can lead to biased or inflated estimates of association. As this analysis was done on prospectively collected data within a trial context with preset procedures rather than routine data, we also minimized the common problem of missing data and information bias.

There are some limitations of the study. First, although the sample size is small, the prevalence of VF on bPI we found is in line with other published studies. The inclusion of whole-genome sequencing data, albeit from 9 patients, is unique to this cohort. Second, the study was nested in a randomized community trial and as such the patients may not be truly reflective of the general HIV population, given the intense monitoring and provision of counseling and adherence interventions that may impact their attitude toward health and promote more successful treatment outcomes. We did not measure drug levels to assess pharmacokinetic drug–drug interactions or adherence before a clinical visit. Finally, 16 (15.8%) patients were included who were already on second-line ART at enrollment, for a median of 2.7 years, representing a potential bias.

There was also heterogeneity in the patient population. We included all participants from the parent study irrespective of study arm, though study arm had no effect on outcome, largely due to poor linkage to care in this setting (Dabis et al, 21st International AIDS Conference 2016, Durban, abstract FRAC0105LB). We included newly diagnosed as well as ART-experienced patients, as both present for first-line therapy under “real-world” programmatic conditions, sometimes with evidence of drug resistance to thymidine analogues arising from prior ART [51] or with evidence of transmitted drug resistance [52].

In conclusion, this study found that second-line PI-based VF was common in this population accessing ART in rural South Africa under trial circumstances but recruited at the population level. Further research is needed to understand the mechanisms behind VF of bPI ART in TB-coinfected patients. Novel approaches to optimize second-line ART in resource-limited settings are still urgently needed as this population is likely to grow rapidly, owing to the WHO 2015 guidelines to test and treat all people living with HIV [53].

## Supplementary Material

Supplementary Data

## Notes

***Author contributions*** D. C., R. K. G., F. D., D. P., and C. I. designed the study; R. G., D. C., N. O., C. I., F. D., A. C., and D. P. collected and analyzed the data; D. C. and R. G. wrote the first draft; A. D. and T. de O. performed experiments and analyzed data.

***Financial support.*** The French National Agency for AIDS and Viral Hepatitis Research (ANRS) is the sponsor and co-funder of the trial. Research discussed in this publication has been co-funded by 3ie with support from the Bill & Melinda Gates Foundation. The Deutsche Gesellschaft für Internationale Zusammenarbeit co-funded the first part of the trial. The trial is conducted with the support of Merck & Co and Gilead Sciences, which provided the Atripla drug supply. The Africa Health Research Institute for Population Health receives core funding from the Wellcome Trust, which provides the platform for the population- and clinic-based research at the Centre. D. C. is funded by a British Infection Association fellowship and R. G. is funded by a Wellcome Senior Fellowship (WT108082AIA).

***Potential conflicts of interest*** All authors: No potential conflicts. All authors have submitted the ICMJE Form for Disclosure of Potential Conflicts of Interest. Conflicts that the editors consider relevant to the content of the manuscript have been disclosed.

## APPENDIX

**Appendix Table 1.** ANRS 12249 Treatment as Prevention Study Group (as of March 2016)

Name	Role	Affiliation
Investigators		
François Dabis	Co-PI (France)	- Université. Bordeaux, ISPED, Centre Inserm U1219 Bordeaux Population Health, Bordeaux, France
		- INSERM, ISPED, Centre Inserm U1219 Bordeaux Population Health, Bordeaux, France
Deenan Pillay	Co-PI (South Africa)	- Africa Centre for Population Health, University of KwaZulu- Natal, South Africa
		- Faculty of Medical Sciences, University College London, United Kingdom (UK)
Marie-Louise Newell	Co-PI (United Kingdom)	- Africa Centre for Population Health University of KwaZulu- Natal, South Africa
		-Faculty of Medicine, University of Southampton, UK
Coordinators		
Collins Iwuji	Trial Coordinator and HIV Clinician (South Africa)	- Africa Centre for Population Health, University of KwaZulu- Natal, South Africa
		- Research Department of Infection and Population Health, University College London, UK
Joanna Orne-Gliemann	Trial Coordinator (France)	- Univ. Bordeaux, ISPED, Centre Inserm U1219 Bordeaux Population Health, Bordeaux, France
		- INSERM, ISPED, Centre Inserm U1219 Bordeaux Population Health, Bordeaux, France
Study team		
Kathy Baisley	Statistics	- Department of Global Health and Development, London School of Tropical Medicine and Hygiene
		- Africa Centre for Population Health, University of KwaZulu- Natal, South Africa
Till Bärnighausen	Health economics	- Africa Centre for Population Health, University of KwaZulu- Natal, South Africa
		- Dept of Global Health & Population, Harvard School of Public Health, Harvard Univ. Boston, USA
Eric Balestre	Epidemiology and Biostatistics	- Univ. Bordeaux, ISPED, Centre Inserm U1219 Bordeaux Population Health, Bordeaux, France
		- INSERM, ISPED, Centre Inserm U1219 Bordeaux Population Health, Bordeaux, France
Sylvie Boyer	Health economics	- INSERM, UMR912 (SESSTIM), Marseille, France
		- Aix Marseille Université, UMR_S912, IRD, Marseille, France
		- ORS PACA, Observatoire Régional de la Santé Provence- Alpes-Côte d’Azur, Marseille, France
Alexandra Calmy	Adult Medicine	- Service des maladies infectieuses, Hôpital Universitaire de Geneve, Genève, Switzerland
Vincent Calvez	Virology	- Department of virology, Hôpital Pitié-Salpétrière, Paris, France
Anne Derache	Virology	- Africa Centre for Population Health, University of KwaZulu- Natal, South Africa
Hermann Donfouet	Statistics/Economist	- INSERM, UMR912 (SESSTIM), Marseille, France
		- Aix Marseille Université, UMR_S912, IRD, Marseille, France
		- ORS PACA, Observatoire Régional de la Santé Provence- Alpes-Côte d’Azur, Marseille, France
Rosemary Dray-Spira	Social sciences	- INSERM U1018, CESP, Epidemiology of Occupational and Social Determinants of Health, Villejuif, France
		- University of Versailles Saint-Quentin, UMRS 1018, Villejuif, France
Jaco Dreyer	Data management	- Africa Centre for Population Health, University of KwaZulu- Natal, South Africa
Andrea Grosset	Statistics	- INSERM, UMR912 (SESSTIM), 13006, Marseille, France
		- Aix Marseille Université, UMR_S912, IRD, Marseille, France
		- ORS PACA, Observatoire Régional de la Santé Provence- Alpes-Côte d’Azur, Marseille, France
Kobus Herbst	Data management	- Africa Centre for Population Health, University of KwaZulu- Natal, South Africa
John Imrie	Social sciences	- Africa Centre for Population Health, University of KwaZulu- Natal, South Africa
		- Centre for Sexual Health and HIV Research, Research Department of Infection and Population, Faculty of Population Health Sciences, University College London, London, UK
Joseph Larmarange	Social sciences	- CEPED (Centre Population & Développement-UMR 196- Paris Descartes/INED/IRD), IRD (Institut de Recherche pour le Développement), Paris, France.
		- Africa Centre for Population Health, University of KwaZulu- Natal, South Africa
France Lert	Social Sciences	- INSERM U1018, CESP, Epidemiology of Occupational and Social Determinants of Health, Villejuif, France
		- University of Versailles Saint-Quentin, UMRS 1018, Villejuif, France
Thembisa Makowa	Field operations	- Africa Centre for Population Health, University of KwaZulu- Natal, South Africa
Anne-Geneviève Marcelin	Virology	- Department of virology, Hôpital Pitié-Salpétrière, Paris, France
Nuala McGrath	Epidemiology/Social sciences	- Faculty of Medicine and Faculty of Human, Social and Mathematical Sciences, University of Southampton, UK - Africa Centre for Population Health, University of KwaZulu- Natal, South Africa
		- Research Department of Infection and Population Health, University College London, UK
Nonhlanhla Okesola	Nurse manager	- Africa Centre for Population Health, University of KwaZulu- Natal, South Africa
Tulio de Oliveira	Bioinformatics	- Africa Centre for Population Health, University of KwaZulu- Natal, South Africa
Delphine Perriat	Epidemiology/social sciences	- Univ. Bordeaux, ISPED, Centre Inserm U1219 Bordeaux Population Health, Bordeaux, France
		- INSERM, ISPED, Centre Inserm U1219 Bordeaux Population Health, Bordeaux, France
Melanie Plazy	Epidemiology/social sciences	- Univ. Bordeaux, ISPED, Centre Inserm U1219 Bordeaux Population Health, Bordeaux, France
		- INSERM, ISPED, Centre Inserm U1219 Bordeaux Population Health, Bordeaux, France
Camelia Protopopescu	Statistics/Economist	- INSERM, UMR912 (SESSTIM), Marseille, France
		- Aix Marseille Université, UMR_S912, IRD, Marseille, France
		- ORS PACA, Observatoire Régional de la Santé Provence- Alpes-Côte d’Azur, Marseille, France
Luis Sagaon-Teyssier	Health economics	- INSERM, UMR912 (SESSTIM), Marseille, France
		- Aix Marseille Université, UMR_S912, IRD, Marseille, France
		- ORS PACA, Observatoire Régional de la Santé Provence- Alpes-Côte d’Azur, Marseille, France
Bruno Spire	Health economics	- INSERM, UMR912 (SESSTIM), 13006, Marseille, France
		- Aix Marseille Université, UMR_S912, IRD, Marseille, France
		- ORS PACA, Observatoire Régional de la Santé Provence- Alpes-Côte d’Azur, Marseille, France
Frank Tanser	Epidemiology and Biostatistics	- Africa Centre for Population Health, University of KwaZulu- Natal, South Africa
Rodolphe Thiébaut	Epidemiology and Biostatistics	- Univ. Bordeaux, ISPED, Centre Inserm U1219 Bordeaux Population Health, Bordeaux, France
		- INSERM, ISPED, Centre Inserm U1219 Bordeaux Population Health, Bordeaux, France
Thierry Tiendrebeogo	Epidemiology and Biostatistics	- Univ. Bordeaux, ISPED, Centre Inserm U1219 Bordeaux Population Health, Bordeaux, France
		- INSERM, ISPED, Centre Inserm U1219 Bordeaux Population Health, Bordeaux, France
Thembelihle Zuma	Psychology/Social sciences	- Africa Centre for Population Health, University of KwaZulu- Natal, South Africa
Scientific advisory board		
Chair: Bernard Hirschel (Switzerland)		
International experts		
Xavier Anglaret (Ivory Coast)		
Hoosen Cooavdia (South Africa)		
Alpha Diallo (France)		
Bruno Giraudeau (France)		
Jean-Michel Molina (France)		
Lynn Morris (South Africa)		
François Venter (South Africa)		
Sibongile Zungu (South Africa)		
Community representatives		
Eric Fleutelot (France)		
Eric Goemaere (South Africa)		
Calice Talom (Cameroon)		
Sponsor representatives (ANRS)		
Brigitte Bazin		
Claire Rekacewicz		
Pharmaceutical company representatives		
Golriz Pahlavan-Grumel (MSD)		
Alice Jacob (Gilead)		
Data safety and monitoring board		
Chair: Patrick Yeni (France)		
Sinead Delany-Moretlwe (South Africa)		
Nathan Ford (South Africa)		
Catherine Hankins (Netherlands)		
Helen Weiss (UK)		
