# Supplementary material for: Virological Outcomes of Second-line Protease Inhibitor–Based Treatment for Human Immunodeficiency Virus Type 1 in a High-Prevalence Rural South African Setting: A Competing-Risks Prospective Cohort Analysis
Source: Clin Infect Dis. 2017 Mar 13;64(8):1006–16. doi: 10.1093/cid/cix015 (PMC5439490; doi:10.1093/cid/cix015)
Supplement: Supplementary Data [file cix015_Supplementary_Data.zip › 83842R1_CID_Supplementary_material.pdf]

Supplementary material

Methods:

The TasP trial was implemented on the background of a public ART program which started in 2004. All HIV-positive individuals 16 years and above were eligible for enrolment in the trial irrespective of their ART status as there was already an established public ART programme in the trial communities. However those who self-reported to be HIV-positive but ART naive or newly diagnosed were offered ART based on whether their community was randomised to the intervention or control arm. Those randomised\ to the intervention arm were offered ART regardless of CD4 count while those randomised to the control arm were offered ART according to South African guidelines.

As part of the TasP trial, all participants identified as HIV-positive through home-based HIV testing were referred to the TasP clinics within their cluster area, i.e located within 5 km of their homes. Participants were seen monthly in the clinics. In 2011, the ART coverage within the sub-district was estimated at 37% of all HIV-infected adults[23]. Hence TasP enrolled both ART-experienced and newly identified HIV-positive individuals as long as they were resident within the TasP clusters. The trial started recruiting in March 2012 in Hlabisa sub-district, Umkhanyakude district, Northern KwaZulu-Natal.

ART was based on the national treatment guidelines. Between 2004 and 2010, first-line ART regimens were stavudine (d4T) or zidovidine (AZT) + lamivudine (3TC) + efavirenz (EFV) or nevirapine (NVP)[24]. In 2010 tenofovir (TDF) became available, substituting for D4T or AZT[25]. The second-line ART regimen consisted of boosted lopinavir/ritonavir (LPV/r) + didanosine (ddI) + AZT. This was changed to LPV/r + 3TC/FTC + AZT or TDF in 2010 [24, 25].

Age at second-line treatment initiation was categorised into two groups:16-35 and >35 years in order to generate equal numbers in each group. Relationship status was categorised into three groups: single, married and widowed. Highest education level attained was categorised into three groups: primary or less, some secondary, completed secondary. Asset category was based on a validated household asset ownership index score and categorised into three groups: low, medium and high. The World Health Organization (WHO) HIV clinical stage was documented at initiation of second-line

treatment. The nadir CD4 prior to first-line ART count was categorised into two groups according to perceived risk of opportunistic infections: 0-100 and >100 cell/$mm^3$. The number of clinic visits per year of second-line treatment was categorised into two groups: 0-11 and ≥12 visits per year in order to generate equal numbers in each group. Adherence to treatment was estimated by the median pill count in the duration on second-line treatment; this figure was derived from the difference between tablets dispensed and returned, expressed as a percentage of the number expected to be taken. TB treatment was defined as receipt of a course of rifampicin containing antituberculous therapy at any time between 6 months before and until six months after VF. Rifabutin was not available in the TasP trial.

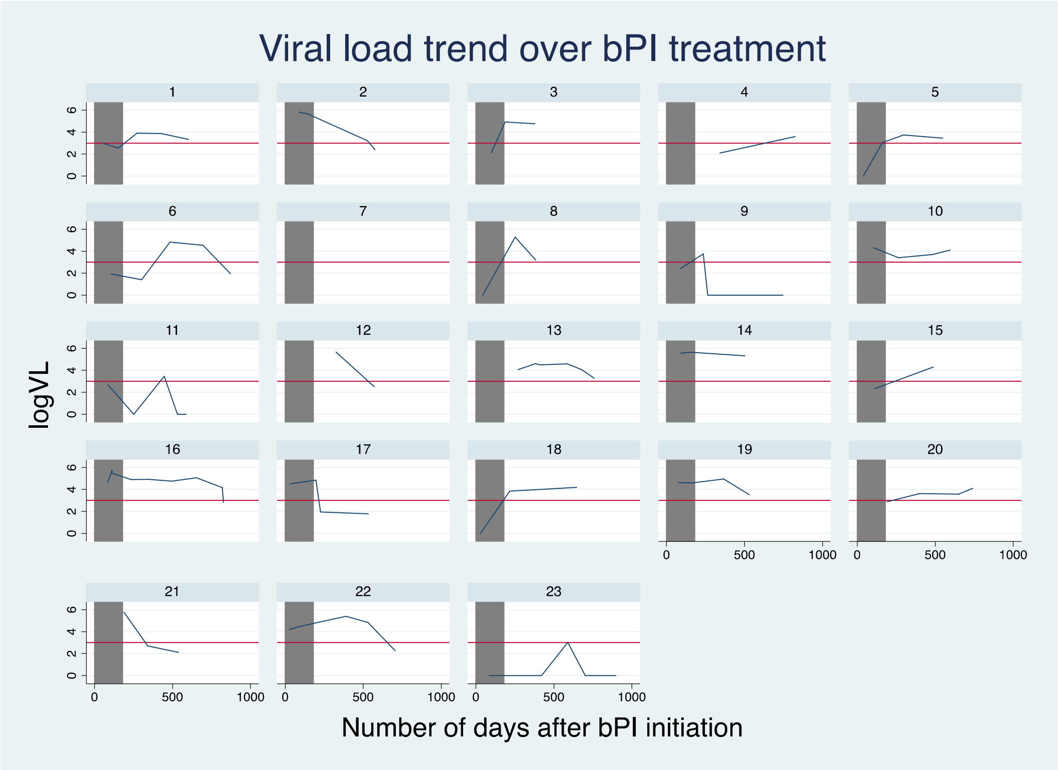


Supplementary Figure 1: the trend in Viral load over the course of bPI ART in the 23 patients with VF. The grey bar indicates the 6-month duration from initiation of bPI ART. The red line indicates the cut off for VF of VL>1000 copies/mL (or log $_{10}$ VL of 3). Numbers 4,13 and 20 are the 3 inidividuals with bPI-VF at entry into the study. Numbers 1 (8), 2 (1), 3 (7), 5 (6), 7 (9), 8 (3), 14 (5), 18 (2) and 19 (4) had next generation sequencing performed on at least one of either their first or second-line failure sample. The number in bracket indicates the corresponding participant ID in Table 3.

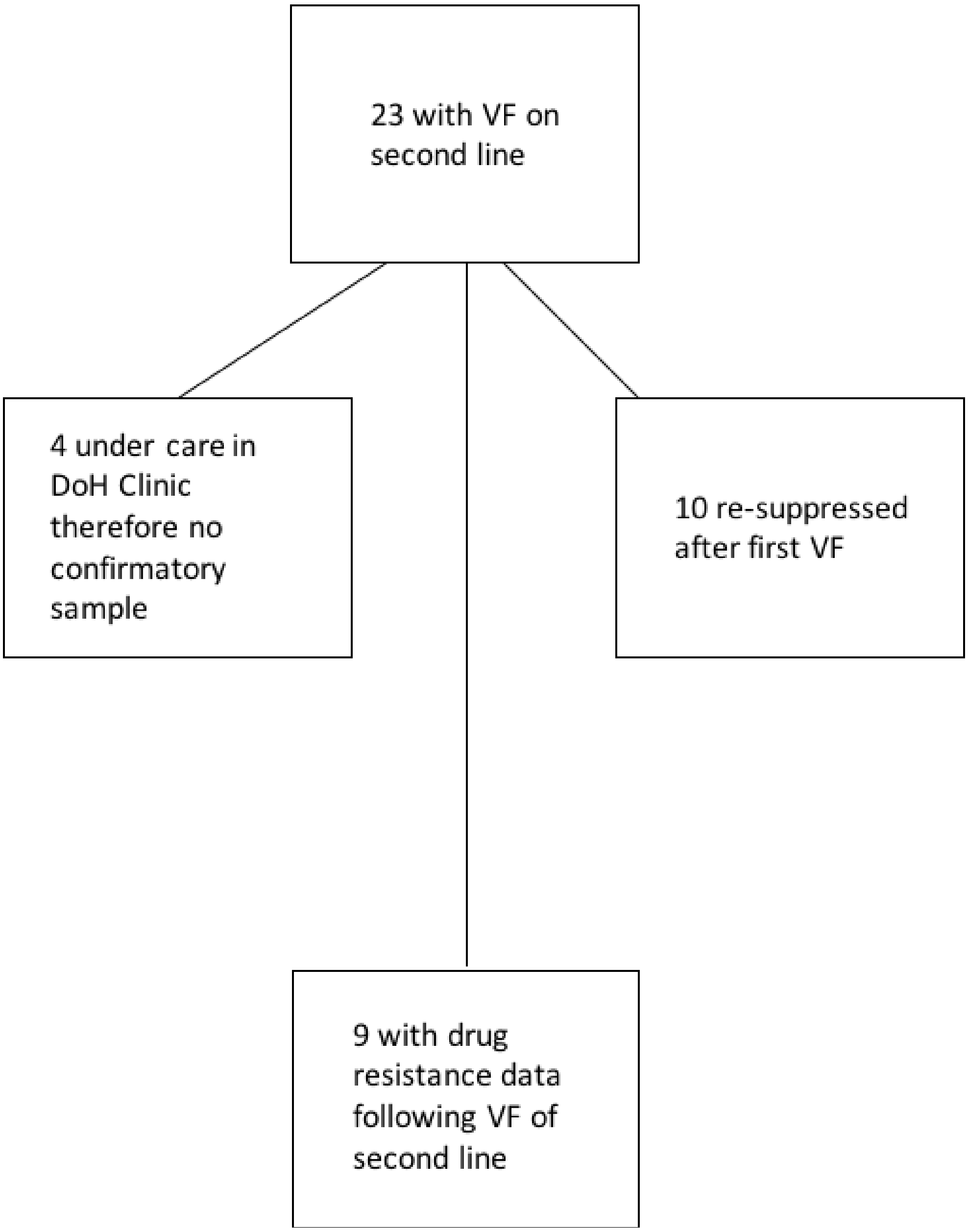


Supp Figure 2: Flow diagram showing 23 patients with VF on a single sample and those with subsequent drug resistance data.
